#  miR-495 inhibits the growth of fibroblasts in hypertrophic scars

**DOI:** 10.18632/aging.101965

**Published:** 2019-05-14

**Authors:** Bingyu Guo, Qiang Hui, Zhishan Xu, Peng Chang, Kai Tao

**Affiliations:** 1 Reconstructive and Plastic Surgery, General Hospital of Northern Theater, PLA, Shenyang, P.R. China

**Keywords:** FAK, hypertrophic scar, miR-495, proliferation, apoptosis

## Abstract

Noncoding RNAs are known to be importantly involved in a variety physiological and pathophysiolgical processes. Their role in the pathogenesis of hypertrophic scars remains unclear, however. After preliminary screening of the microRNA (miRNA) gene expression profiles, we explored the role of miR-495 in the development of hypertrophic scar by comparing expression of miR-495 and focal adhesion kinase (FAK) between hypertrophic scar and normal skin tissue. We also used 3-(4,5-dimethyl-2-thiazolyl)-2,5-diphenyl-2-H-tetrazolium bromide and annexin V-fluorescein isothiocyanate/propidium iodide assays to assess the effect of miR-495 on the proliferation and apoptosis in human hypertrophic scar fibroblasts. Western blotting and real-time polymerase chain reaction were used to evaluate expression of miR-495, FAK, and related proteins in the FAK pathway. Our findings show that miR-495 inhibits FAK and its downstream mediators in vitro and vivo, and suggest that miR-495 may be a useful therapeutic target for the treatment of hypertrophic scar.

## Introduction

A scar is the mark left by wound healing and is the final result of tissue repair and healing. In some individuals, abnormalities in the repair process can lead to excessive tissue proliferation and hypertrophic scarring. Hypertrophic scars protrude from the skin and are irregular in shape, uneven in height, reddish due to hyperemia, and strong/tough in texture [1]. In addition, they may burn or itch, and those symptoms may be aggravated when the ambient temperature increases, during emotional excitement, or when eating spicy or irritating food. Because hypertrophic scars can cause serious deformities and other adverse cosmetic effects, they bring patients mental anguish and also pose a financial burden. But although these scars have long been a common clinical problem addressed by surgeons, especially plastic surgeons, the mechanism underlying formation of hypertrophic scars is not yet fully understood [2].

miRNAs are involved in regulating cell proliferation, differentiation and apoptosis. In addition, miRNAs can act to exacerbate or inhibit the pathogenesis and progression of various diseases, including cancer and fibrosis. For example, miR-495 reportedly inhibits the growth and metastasis of melanoma and gastric, colon, breast and liver cancers [3-9]. It also inhibits high glucose-induced inflammation as well as the differentiation cardiac cells and the cardiac accumulation of extracellular matrix [10]. On the other hand, there are no reports on whether miR-495 plays a role in hypertrophic scarring.

Focal adhesion kinase (FAK) appears to be involved in the process of wound healing [11], playing a key role in regulating fibrosis. This makes FAK a potential therapeutic target in fibrotic diseases [12]. In the present study, we compared expression of miRNAs in hypertrophic scar tissue with that in normal skin epidermis. Or particular interest was the level of miR-495 expression and its effect on the growth of fibroblasts. Our findings indicate that miR-495 can inhibit fibroblast growth by inhibiting FAK.

## RESULTS

### Differential expression profile of miRNAs in hypertrophic scar tissues

**Figure 1:**
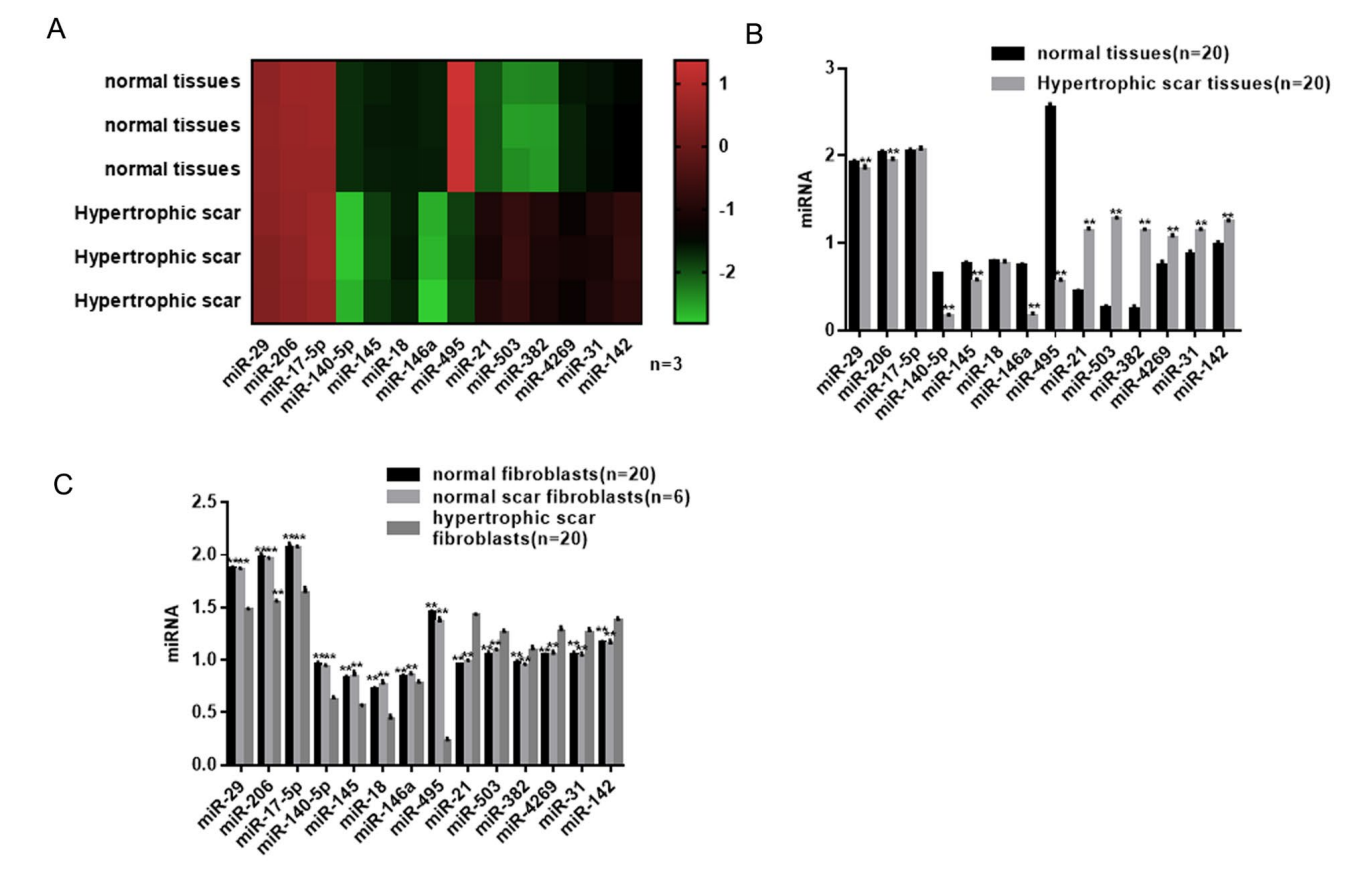
**Differential expression profile of miRNAs in hypertrophic scar tissues.** (**A**) miRNAs differentially expressed in hypertrophic scar and normal tissues were detected using a gene microarray. (**B**) Expression levels of miRNAs in hypertrophic scar and normal tissues were detected using real-time PCR. ** P< 0.01 vs. normal tissues (**C**) Expression levels of miRNAs in hypertrophic scar fibroblasts, normal scar fibroblasts and normal fibroblasts were detected using real-time PCR. ** P< 0.01 vs. hypertrophic scar fibroblasts.

The miRNA microarray revealed six downregulated (*P*<0.05) and six upregulated miRNAs (*P*<0.05) in hypertrophic scar tissues compared with normal tissues (Figure 1A). Using real-time PCR, we found that among the differentially expressed miRNAs, miR-495 showed the change (Figure 1B). Expression of miR-495 was confirmed to be significantly lower in HSFs than normal scar fibroblasts and normal skin fibroblasts (Figure 1C).

### Relationship between miR-495 and FAK

**Figure 2:**
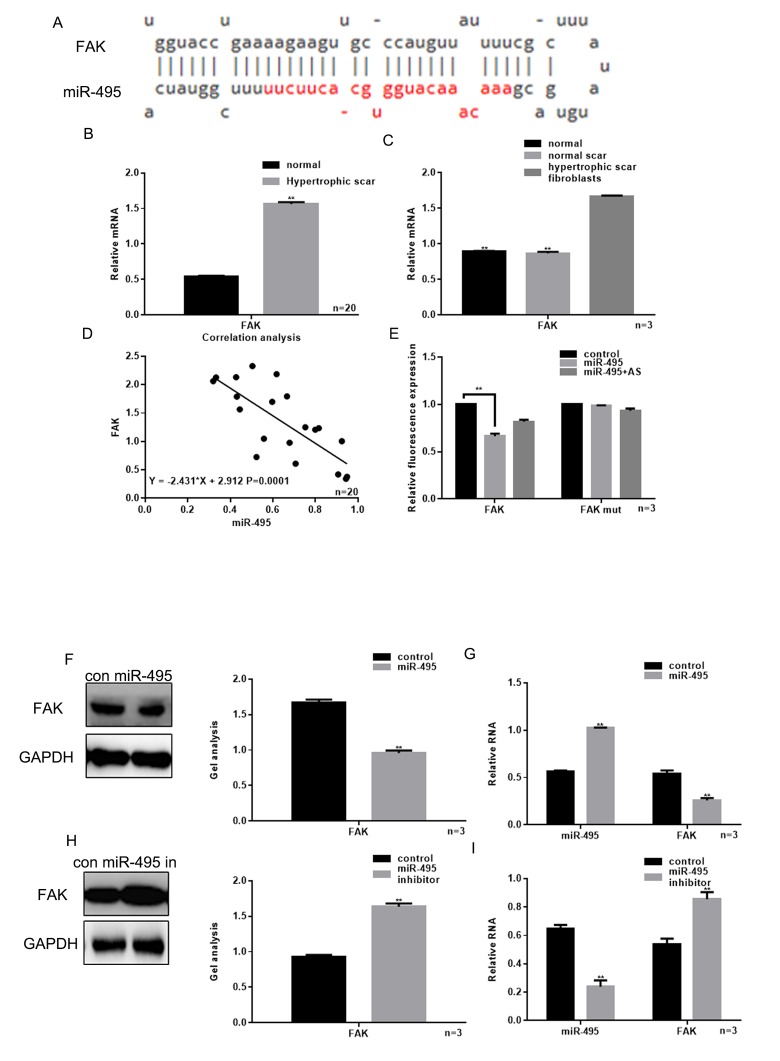
**Relationship between miR-495 and FAK**. (**A**) miRDB predicted that miR-495 specifically combined with FAK mRNA. (**B**) Expression of FAK in hypertrophic scar tissues and normal tissues were detected using real-time PCR. ** P< 0.01 vs. normal tissues. (**C**) Levels of FAK expression in hypertrophic scar fibroblasts, normal scar fibroblasts and normal fibroblasts were detected using real-time PCR. ** P< 0.01 vs. fibroblasts, *## P*< 0.01 vs. scar fibroblasts. (**D**) Correlation between expression of miR-495 and FAK in hypertrophic scar. (**E**) Interaction between miR-495 and the FAK 3’-UTR was tested in luciferase reporter assays. Data are presented as the mean ± SEM. ** P< 0.01 vs. control. (**F**, **G**) Western blotting and real-time PCR showing that when miR-495 is overexpressed, FAK expression is downregulated. Data are presented as the mean ± SEM. ** P< 0.01 vs. control. (H, I) Western blotting and real-time PCR analysis showing that when miR-495 is repressed, FAK expression is upregulated. Data are presented as the mean ± SEM. ** P< 0.01 vs. control.

miRDB software was demonstrated that miR-495 could target the 3’UTR of FAK (Figure 2A). Results showed that there was a higher expression of FAK in hypertrophic scar tissues, compared with normal tissues (Figure 2B). Real-time PCR showed that the expression of FAK in HSFs was higher than normal and normal scar fibroblasts (Figure 2C).

We also observed that there was a negative correlation between the levels of miR-495 and FAK expression in hypertrophic scar tissue (Figure 2D). Luciferase reporter assays showed that when HSFs were co-transfected with FAK and miR-495, FAK-driven luciferase activity was decreased as compared to control (*P*<0.05). On the other hand, when HSFs were co-transfected with miR-495 and FAK mut or with FAK and miR-495 AS there was not a significant change from control (Figure 2E). Consistent with those findings, western blotting and real-time PCR showed that the expression of FAK was downregulated by miR-495 and upregulated by miR-495 inhibitor (Figure 2F-I).

### miR-495 inhibits the growth of HSFs

**Figure 3:**
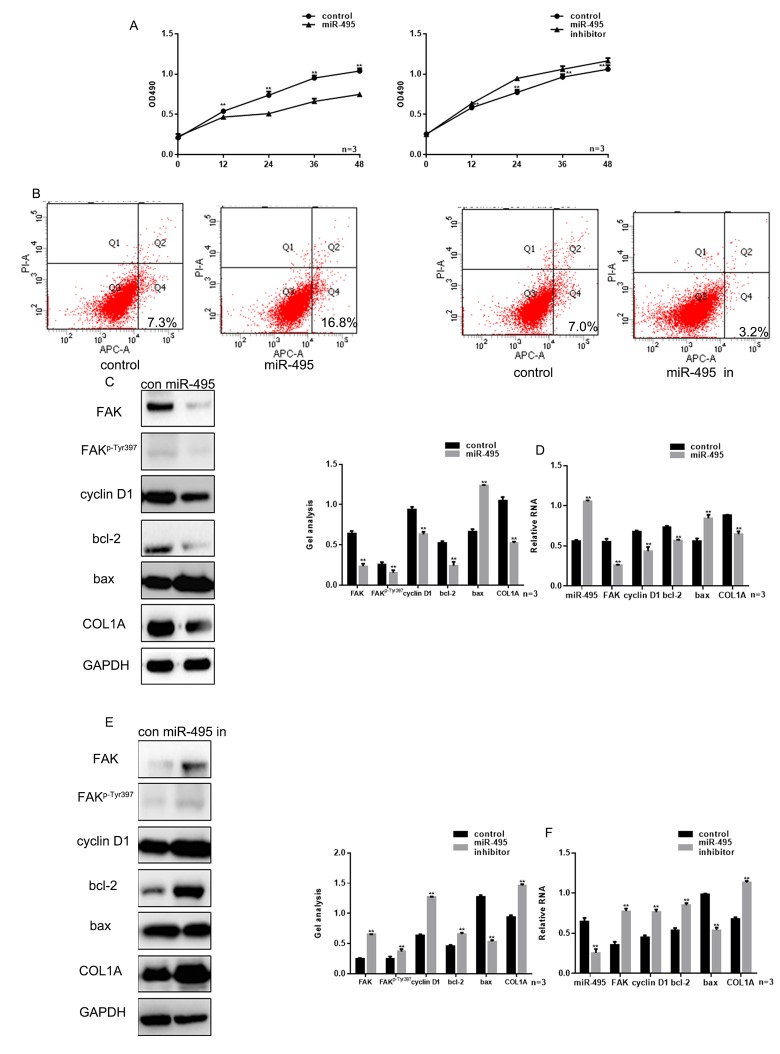
**miR-495 inhibits HSF growth.** (**A**) MTT assay showing that following transfection with miR-495 mimic/inhibitor, HSF proliferation is repressed/enhanced. Data are presented as the mean ± SEM. ** P< 0.01 vs. control. (**B**) AV-PI assay showing that following transfection with miR-495 mimic/inhibitor, HSF apoptosis is enhanced/repressed. (**C**, **D**) Following transfection of HSFs with miR-495 mimic, expression of FAK, FAK^p-Tyr397^, cyclin D1, bcl-2, bax and COL1A were detected using western blotting and real-time PCR. Data are presented as the mean ± SEM. ** P< 0.01. (**E**, **F**) Following downregulation of miR-495, expressions of FAK, FAK^p-Tyr397^, cyclin D1, bcl-2, bax and COL1A were detected using western blotting and real-time PCR. Data are presented as the mean ± SEM. ** P< 0.01.

We next used MTT assays to evaluate proliferation HSFs transfected with miR-495 mimic or inhibitor into HSFs. The result showed that miR-495 significantly inhibited the HSF growth and that miR-495 inhibitor had the opposite effect (Figure 3A). Annexin V-fluorescein isothiocyanate/propidium iodide (AV-PI) assays showed that the overexpression of miR-495 promoted HSF apoptosis, while miR-495 inhibitor again had the opposite effect (Figure 3B). Cyclin D1, bcl-2 and bax are important proteins involved in regulation of the cell cycle and apoptosis, and are also involved in the FAK pathway [13]. Hypertrophic scarring is closely related to expression of genes encoding mediators involved in cytoskeletal movement, including COL1A, which is often highly expressed in those tissues. Notably, COL1A expression is regulated in part via the FAK signaling pathway [11]. Moreover, using western blotting and real-time PCR we found that miR-495 significantly inhibited expression of FAK, FAK^p-Tyr397^, cyclin D1, bcl-2 and COL1A, but promoted expression of bax (Figure 3C-F).

### FAK is crucial to the inhibition of miR-495 on HSFs growth

**Figure 4:**
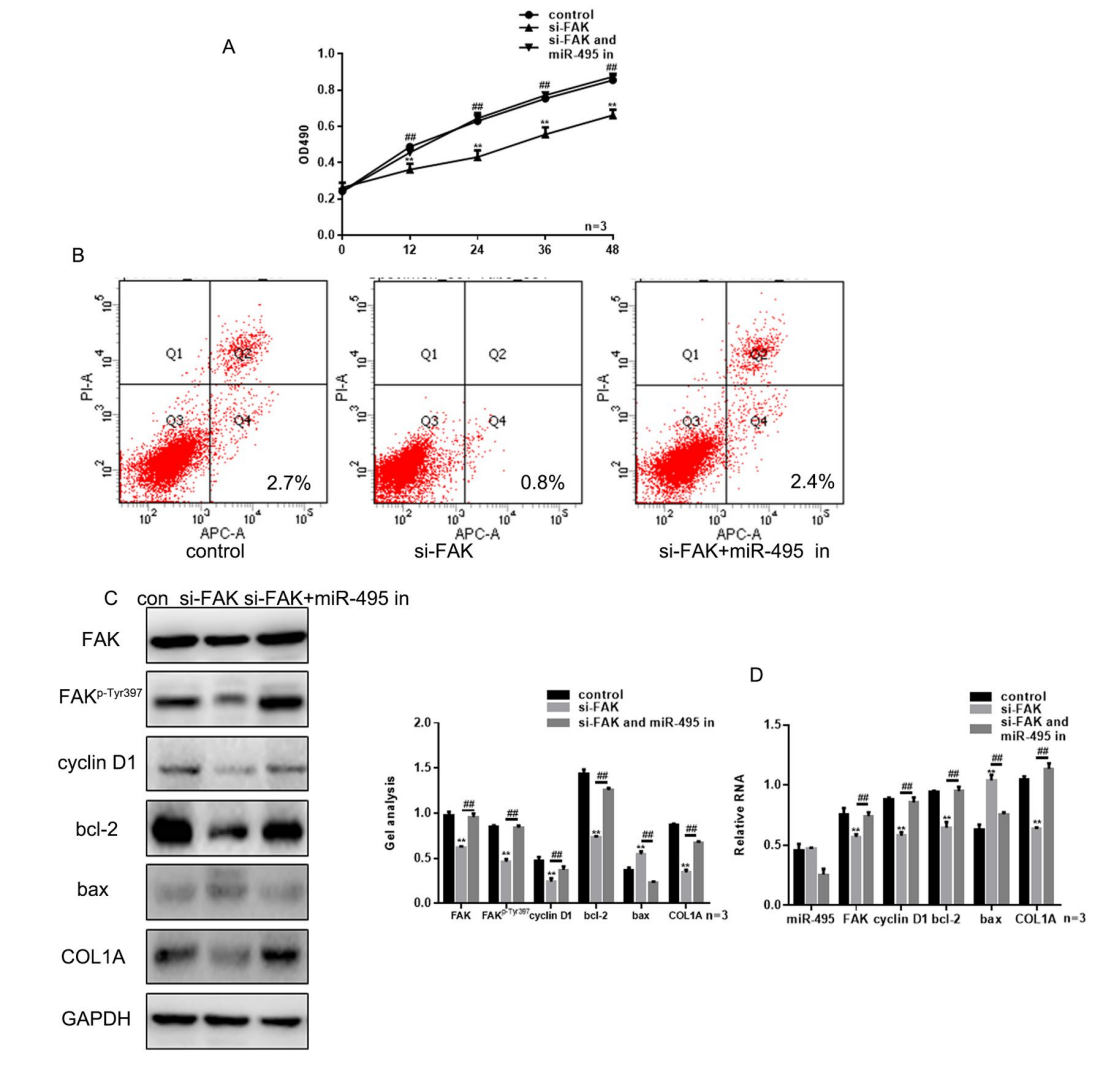
**FAK is crucial to the inhibitory effect of miR-495 on HSFs growth.** (**A**) MTT assays showing the effects of si-FAK and miR-495 inhibitor on HSF proliferation. Data are presented as the mean ± SEM. ** P< 0.01 si-FAK vs. control, *## P*< 0.01 si-FAK+miR-495 inhibitor vs. si-FAK. (**B**) AV-PI assays showing the effects of si-FAK and miR-495 inhibitor on HSF apoptosis. (**C**, **D**) Following transfection of HSFs with si-FAK or si-FAK plus miR-495 inhibitor, expression of FAK, FAK^p-Tyr397^, cyclin D1, bcl-2, bax and COL1A were detected using western blotting and real-time PCR. Data are presented as the mean ± SEM. ** P< 0.01 si-FAK vs. control, *## P*< 0.01 si-FAK+miR-495 inhibitor vs. si-FAK.

**Figure 5:**
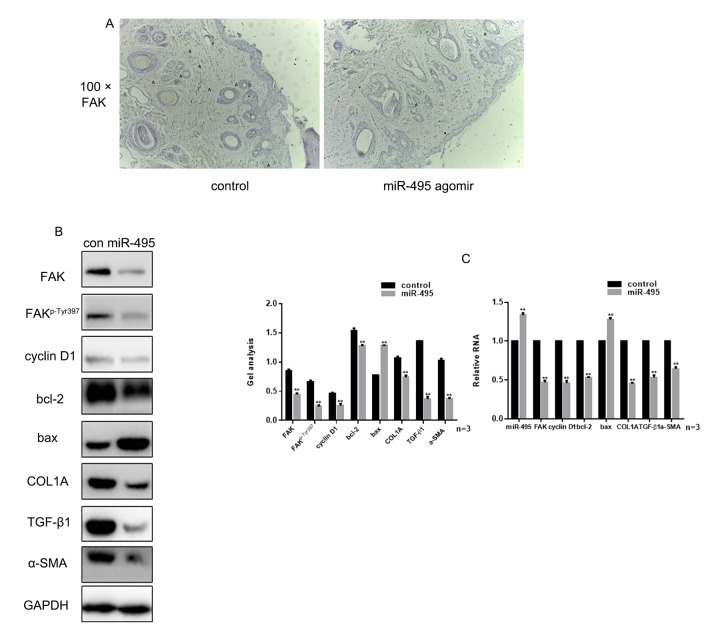
**miR-495 inhibits FAK in vivo** (**A**) Comparison of initial area, average healing time and scar area after healing in each group. (**B**) Immunohistochemical staining showing expression of FAK in the control and miR-495 agomir groups (magnification: 100×). (**C**, **D**) Expression of FAK, FAK^p-Tyr397^, cyclin D1, bcl-2, bax, COL1A, TGF-β1 and α-SMA were detected using western blotting and real-time PCR. Data are presented as the mean ± SEM. ** P< 0.01

Knocking down FAK expression using si-FAK significantly suppressed HSF growth (Figure 5A-B). However, treatment with miR-495 inhibitor counteracted the inhibition of HSF proliferation and promotion of their apoptosis induced by silencing FAK (Figure 4A-B). This likely reflects the fact that in HSFs treated with both si-FAK and miR-495 inhibitor, FAK levels were markedly higher than in cells treated with si-FAK alone, and expression of related proteins exhibited the same trend (Figure 4C-D).

### miR-495 inhibits FAK in vivo

Examination of wound healing in vivo showed that there was no significant difference in the initial wound area between groups, and there was no difference in the average healing times. However, the scar area was significantly smaller in the miR-495 agomir group was smaller than in the control group (Table 1). Immunohistochemical analysis showed that overexpression of miR-495 inhibited expression of FAK in vivo (Figure 5A). In addition, western blotting and real-time PCR showed that the expression of FAK, FAK^p-Tyr397^, cyclin D1, bcl-2 and COL1A was inhibited, while expression of bax was promoted, in tissue overexpressing miR-495 (Figure 5B-C).

**Table 1:** the primers of RNAs.

Name	Forward primer(5'->3')	Reverse primer(5'->3')
miR-495	ACACUCAAAACAAACAUG	GUGCACUUCUUGAGAGUACA
miR-29	ACACUCAUAGCACCAUC	UGAAAUCGGUUAGAGAGUACA
miR-206	ACACUCAUGGAAUGUAAG	GAAGUGUGUGGGAGAGUACA
miR-17-5p	ACACUCACAAAGUGCUUAC	AGUGCAGGUAGGAGAGUACA
miR-140	ACACUCACAGUGGUUUUAC	CCUAUGGUAGGAGAGUACA
miR-145	ACACUCAGUCCAGUUUUC	CCAGGAAUCCCUGAGAGUACA
miR-18	ACACUCAUAAGGUGCAUC	UAGUGCAGAUAGGAGAGUACA
miR-146a	ACACUCAUGAGAACUGAA	UUCCAUGGGUUGAGAGUACA
miR-21	ACACUCAUAGCUUAUCAG	ACUGAUGUUGAGAGAGUACA
miR-503	ACACUCAUAGCAGCGGGAA	CAGUUCUGCAGGAGAGUACA
miR-382	ACACUCAGAAGUUGUUCG	UGGUGGAUUCGGAGAGUACA
miR-4269	ACACUCAGCAGGCACAG	ACAGCCCUGGCGAGAGUACA
miR-31	ACACUCAAGGCAAGAUG	CUGGCAUAGCUGAGAGUACA
miR-142	ACACUCAUGUAGUGUUUC	CUACUUUAUGGAGAGAGUACA
U6	CUCGCUUCGGCAGCACA	ACGCUUCACGAAUUUGCGU
H FAK	GACAGGGAGGATGGAAGTC	TACTCTTGCTGGAGGCT
H cyclin D1	CCAACCTCCTCAACGACC	TGGCACAGAGGGCAACGAAG
H bcl-2	CTTTGAGTTCGGTGGGGTC	TGCATATTTGTTTGGGGCAGG
H bax	TCCACCAAGAAGCTGAGCG	GTCCAGCCCATGATGGTTC
H COL1A	AGTGGTTTGGATGGTGCCA	GCACCATCATTTCCACGAGC
H GAPDH	CATCCCTTCTCCCCACAC	GTCCCAGGGCTTTGATTTG
R FAK	CTTGGACGCTGTATTGGAG	CTGTTGCCTGTCTTCTGGAT
R cyclin D1	GGAGCAGAAGTGCGAAGA	GGGTGGGTTGGAAATGAA
R bcl-2	CTGGTGGACAACATCGCTC	GGTCTGCTGACCTCACTTG
R bax	GCGAATTGGAGATGAACTG	GTGAGCGAGGCGGTGAGGAC
R COL1A	GCTCGTGGAAATGATGGTG	CCTCGCTTTCCTTCCTCTCC
R TGF-β1	GCTGAACCAAGGAGACGG	GGATCCACTTCCAACCCAGG
R a-SMA	GCTCTGCCTCTAGCACACA	GGCCAGGGCTACAAGTTAAG
R GAPDH	GTATGACTCCACTCACGGC	CTCGCTCCTGGAAGATGG
		

All the reactions were carried out as described previously [31].

## DISCUSSION

MiRNAs play important regulatory roles in many processes; consequently, abnormal expression of miRNA is closely related to the occurrence and development of disease. In recent years, people have begun to notice a relation between miRNA and the occurrence and development of hypertrophic scars [1]. Moreover, key pathological processes involved in hypertrophic scar formation appear similar to those involved keloid formation – i.e., transforming growth factor β signaling, extracellular matrix deposition and fibroblast proliferation and differentiation [1,14]. In that regard, miR-26a and miR-155 inhibit the development of hypertrophic scar fibroblasts [15,16], while miR-519d, miR-222 and miR-6836-3p regulate the viability of fibroblasts [17-19]. It is therefore anticipated that examination of the specific roles of miRNAs in hypertrophic scar formation will reveal biological markers enabling targeted individualized treatment that will improve patients’ clinical symptoms and prognosis.

Using gene chip technology, we found that miR-495 expression was lower in hypertrophic scar tissue than normal scar tissue. miR-495 has been shown to inhibit the biological functions of various tumor cells [6,20-22] and to exert a beneficial effect in some cases of myocardial fibrosis [23]. This suggests miR-495 may play an important role in hypertrophic scars. Consistent with that idea, we found that miR-495 targets FAK expression and that its expression in hypertrophic scar correlates negatively with expression of FAK. Moreover, it was previously shown that FAK plays an important role in the occurrence and development of hypertrophic scars [24]. Deactivation of FAK improves the pathology of hypertrophic scars by suppressing integrin α, TGF-β and α-SMA [11,25]. Our study indicates that FAK is significantly reduced in HSFs overexpressing miR-495 and that overexpression of miR-495 inhibitor has the opposite effect, leading to enhanced FAK expression.

MiR-495 transfection significantly reduced HSF proliferation and promoted their apoptosis. This inhibitory effect may be achieved through the inhibition of FAK signaling by miR-495. An earlier study suggested that FAK may contribute to cell cycle progression by increasing cyclin D1 expression [26]. If so, suppression of FAK could stimulate expression of pro-apoptosis genes, such as BIK, PUMA, BMF, BAX, and MCL-1S, and inhibit the expression of anti-apoptosis genes, such as BCL-2 and BCL-XL [27]. Because it appears that mir-495 can affect HSF growth and target FAK, we suggest that miR-495 may exert its effect on cellular function via downstream FAK-related mediators. Results of western blot and real-time PCR analyses showed that miR-495 suppresses expression of FAK, FAK^p-Tyr397^, cyclin D1 and bcl-2 while promoting expression of bax. One of the main features of hypertrophic scar formation is enhanced collagen synthesis. Our findings indicate that miR-495 also exerts an inhibitory effect on expression of COL1A, which encodes the main component of collagen type 1. It thus appears that miR-495 may exert several effects inhibiting the formation of hypertrophic scars.

To further verify the inhibitory effect of miR-495 on hypertrophic scars, we constructed a rat scar model. Using that model, we found that miR-495 inhibits expression of FAK and related downstream proteins while reducing scar size in vivo.

In summary, the present study revealed that miR-495 plays an important role in hypertrophic scar formation and suggests that miR-495 is a potential therapeutic target for treatment of hypertrophic scars.

## MATERIALS AND METHODS

### Tissue samples and cell lines

Between January 2012 and January 2017 at the General Hospital of Northern Theater. PLA (Shenyang, China), 20 paired hypertrophic scar and normal skin tissue samples were obtained from 20 patients (14 female, 6 male; age range, 19-51 years) during scar excision and flap transplantation, and 6 normal scar tissue samples were obtained from 6 patients (4 female, 2 male; age range, 23-42 years) during flap transplantation. Diagnoses of all patients and tissues were confirmed clinically and pathologically.

Included in the study were patients with no systemic disease. In addition, no patients received any hormonal medication within 3 months before surgery, and none received any local drug, laser or other scar treatment.

Written informed consent was obtained from all patients before the study was performed. The present study was approved by the Ethics Committee of General Hospital of Northern Theater. PLA.

The dermal specimens of hypertrophic scar and normal scar were minced into ~1 mm^3^ fragments then washed in phosphate-buffered saline (PBS). The specimens were then cultured in RPMI-1640 medium (Gibco; Thermo Fisher Scientific, Inc., Waltham, MA USA) supplemented with 10% fetal bovine serum (FBS; Gibco; Thermo Fisher Scientific, Inc., Waltham, MA USA) to obtain hypertrophic scar fibroblasts (HSFs) and normal scar fibroblasts. In addition, cultured human fibroblasts were obtained from the American Type Culture Collection (Manassas, VA, USA) and cultured in RPMI-1640 medium supplemented with 10% FBS. Fibroblasts were cultured in a humidified incubator at 37°C under 5% CO_2_.

### RNA isolation

Total RNA was extracted from tissues, fibroblasts and wound scar tissue using TRIzol reagent (Shenggong, Shanghai, China). The quantity and quality of the extracted RNA were measured with a spectrophotometer (Thermo Scientific, Waltham, MA USA).

### MicroRNA microarray analysis

Aliquots (500 ng) of RNA from three hypertrophic scars and three samples of normal tissue were analyzed using Agilent’s Human miRNA Microarray Release 18.0 (Shanghai Biochip Co., Ltd., China) to detect the miRNAs. Prediction of binding sites between miRNAs and proteins was done using miRDB (http://www.mirdb.org/) and TargetScan ( http://www.targetscan.org).

### Real-time polymerase chain reaction (PCR)

cDNA was obtained using a Reverse Transcription kit (Qiagen GmbH, Hilden, Germany) according to the manufacturer's protocol. PCR was carried out using an iQ5 Real-Time PCR Detection system (Bio-Rad Laboratories, Inc., Hercules, CA, USA). The PCR conditions were: 95°C for 3 min; 40 cycles of 94°C for 20 s, 58°C for 20 s and 72°C for 20 s; and 72°C for 10 min. Expression of FAK, cyclin D1, bcl-2, bax and COL1A mRNA was normalized to expression GAPDH mRNA. U6 small nuclear RNA was used as an internal control for miRNAs. Expression of miR-495 was detected using a Stem-Loop RT-PCR assay as previously described [28,29]. The primer sequences used are listed in Table 2.

**Table 2:** Initial area, average healing time, scar area comparison.

**group**	**N**	**Initial area (mm^2^)**	**average healing time (d)**	**scar area(mm^2^)**
**Control**	10	156.04±3.95	14.23±2.09	29.84±2.13
**miR-495**	10	156.04±3.95	13.98±1.43	20.54±3.97
***P***		0.85	0.77	0.03

### Transfection

HSFs were plated in 6-well plates at a density of 5×10^4^ cells per well. After incubation overnight, the cells were transfected with 50 nM miR-495 mimic, 100 nM miR-495 inhibitor, 50 nM negative control (NC), FAK plasmids, control plasmids, FAK siRNA, or control siRNA (all Shanghai GenePharma, Co., Ltd., Shanghai, China) using Higene (Invitrogen; Beijing, China) according to the manufacturer's protocols. After transfection for 48 h, the HSFs were subjected to following experiments.

### Western blot analyses

Fibroblasts were lysed for 15 min in RIPA buffer (Beyotime Institute of Biotechnology, Shanghai, China) on ice, after which the protein was extracted. In addition, samples of wound scar tissue were ground in liquid nitrogen and mixed with 200 µl of precooled RIPA lysis buffer for 30 min on ice. After removing insoluble materials by centrifugation (13,000 × g, 10 min). The protein was measured then separated using 10% sodium dodecyl sulfate polyacrylamide gel electrophoresis (SDS-PAGE) and transferred to nitrocellulose membranes. The membranes were probed with primary antibodies and then incubated with horseradish peroxidase-conjugated secondary antibodies (Santa Cruz, USA). Relative expression of proteins of interest was quantified and normalized to GAPDH. Gel analysis was performed using Image J software (National Institutes of Health, USA).

### Dual-luciferase reporter assay

To confirm direct target binding, FAK or FAK mutant (FAK mut) 3′-UTR was cloned into a pmiR-RB-ReportTM dual luciferase reporter gene plasmid vector (Guangzhou RiboBio Co., Ltd., Guangzhou, China). HSFs were then co-transfected with 100 ng of FAK or FAK mut and 50 nM miR-495 or miR-495 antisense (miR-495 AS) or its control vector using Higene. After 48 h, luciferase activity was assessed using a Dual-Luciferase Assay System (Promega Corporation, Madison, WI, USA) according to the manufacturer's protocol and normalized to Renilla luciferase activity. The postulated miR-495 target sequence in the FAK 3’UTR was predicted to be 5’-AAGAAGU-3’ by miRDB. The primers used were as follows: for FAK-3’UTR, 5’-CGAGCTCGCCCCTGGCCATTGAACG-3’ (forward) and 5’-CCTCGAGCCCGGCGCCACCTTTTTA-3’ (reverse); for FAK-3’UTR-mutant, 5’-GTCTCAGAAGCTCCTTTTCCTGGAGCCCGTC-3’ (forward) and 5’-CCTGCCTTCCGCCCATATCCACGTGAAGCCAGTTAG-3’(reverse). The sequence of miR-495 AS was 5’-AAGAAGUGCACCAUGUUUGUUU-3’, while the sequence of the negative control RNA was 5’‐GACCUUCAUGUACCUGGCACCG‐3’.

### Cell proliferation assay

3-(4,5-dimethyl-2-thiazolyl)-2,5-diphenyl-2-H-tetrazolium bromide (MTT; Shenggong, Shanghai, China) assays were used to assess cell viability. HSFs were seeded at a density of 1x10^3^ cells/well into the wells of 96-well plates in triplicate and transfected. After transfection for 48 h, 5 µl of MTT was added to each well. After incubation for 4 h at 37°C, the absorbance was measured at 490 nm.

### Cell cycle analysis

HSFs were transfected with miR-495 mimic, miR-495 inhibitor or control for 48 h. The cells were then stained with propidium iodide (100 μg/ml, Qiagen, Beijing, China) for 1 h at 37°C, after which they were fixed in 95% ethanol and analyzed by flow cytometry (BD Pharmingen, Beijing, China).

### Apoptosis assay

HSFs were transfected with miR-495 mimic, miR-495 inhibitor or control for 48 h. The cells then stained with AV-PI (Shenggong, Shanghai, China) for 20 min at room temperature in the dark, after which apoptosis was detected using flow cytometry.

### Rat animal model

All animal experiments were approved by the General Hospital of Northern Theater. PLA Animal Care Committee. All experimental protocols and animal care were performed according to the guidelines for animal experiments of the Institutional Animal Care and Use Committee of General Hospital of Shenyang Military Region. Male Sprague Dawley rats (8 weeks old, average weigh 200 g) were used in the study (Laboratory Animal Center of General Hospital of Shenyang Military Region, Shenyang, China). Twenty male SD rats were randomly divided into experimental and control groups (n=10 each). Rats were first anesthetized by intraperitoneal injection of 7% Chloral Hydrate (0.5 ml/100 g). After depilation of the back using a depilatory agent, two symmetrical deep wounds with an area of 30 mm x 20 mm x 2 mm were made on either side of the spine. The wounds were then treated with 2% diluted iodophor solution after disinfection, and all normal or abnormal exudation was also noted. If abnormal secretion from a wound occurred, the wound was treated for infection.

Chemically synthesized miR-495 agomir or control (RiboBio Co., Ltd., Guangzhou, China) were used to overexpress miR-495. The tissue under the experimental wounds were injected with 100 µl of solution containing 10 nM miR-495 agomir (one side) or control (the other side). The injections were carried out on days 5 and 10 after wounding [30]. After recording was the initial area of the wound, the size of the wound was recorded regularly, as was the time required for wound healing. Twenty days after the wounding, the wound scar tissue was harvested for analysis. The incision was made through the dermis and subcutaneous fascia, exposing the underlying muscle. Gelatin sponge was inserted into the excised wound.

### Immunohistochemistry

Paraffin sections were deparaffinized and rehydrated. Sections were incubated in 2.0% H_2_O_2_/methanol for 30 min to block endogenous peroxidase activity. Slides were autoclaved to unmask antigens. The sections were incubated with primary antibodies against FAK (1:100) overnight at 4°C. After washing, the sections were incubated with biotinylated secondary antibodies and avidin-biotin-peroxidase. The sections were then counterstained with hematoxylin, dehydrated, and cover slipped. Images were obtained at 40× magnification using an Olympus CKX41SF Inverted Phase Contrast Microscope (Nikon Corporation, Tokyo, Japan).

### Statistical analysis

Values are expressed as the mean ± standard deviation of experiments performed in triplicate. Data were analyzed using SPSS version 17.0 (SPSS Inc., Chicago, IL, USA). Groups were compared using Student’s t-test or one-way analysis of variance followed by a post hoc Tukey's test. Statistical significance was defined as P<0.05.

### Ethics approval and consent to participate

Research involving human subjects, human material, or human data, was performed in accordance with the Declaration of Helsinki and was approved by the Research Ethics Committee of General Hospital of Northern Theater. PLA (R20111762).

### Availability of data and material

No restriction on data or material availability.

### Consent for publication

Written informed consent for the publication of all manuscript details was obtained from Bingyu Guo, Qiang Hui, Zhishan Xu, Peng Chang and Kai Tao.
